# Acute Intoxication With Alcohol Reduces Trauma-Induced Proinflammatory Response and Barrier Breakdown in the Lung *via* the Wnt/β-Catenin Signaling Pathway

**DOI:** 10.3389/fimmu.2022.866925

**Published:** 2022-05-18

**Authors:** Laurens Noack, Katrin Bundkirchen, Baolin Xu, Severin Gylstorff, Yuzhuo Zhou, Kernt Köhler, Phatcharida Jantaree, Claudia Neunaber, Aleksander J. Nowak, Borna Relja

**Affiliations:** ^1^ Department of Radiology and Nuclear Medicine, Experimental Radiology, Otto-von-Guericke University, Magdeburg, Germany; ^2^ Trauma Department, Hannover Medical School, Hannover, Germany; ^3^ Institute of Veterinary Pathology, Justus Liebig University Giessen, Giessen, Germany; ^4^ Institute of Experimental Internal Medicine, Otto-von-Guericke University, Magdeburg, Germany

**Keywords:** femur fracture, hemorrhagic shock, inflammation, pulmonary, ethanol

## Abstract

**Background:**

Trauma is the third leading cause of mortality worldwide. Upon admission, up to 50% of traumatized patients are acutely intoxicated with alcohol, which might lead to aberrant immune responses. An excessive and uncontrolled inflammatory response to injury is associated with damage to trauma-distant organs. We hypothesize that, along with inflammation-induced apoptosis, the activation of the Wnt/β-catenin signaling pathway would cause breakdown of the lung barrier and the development of lung injury after trauma. It remains unclear whether ethanol intoxication (EI) prior to trauma and hemorrhagic shock will attenuate inflammation and organ injury.

**Methods:**

In this study, 14 male C57BL/6J mice were randomly assigned to two groups and exposed either to EtOH or to NaCl as a control by an oral gavage before receiving a femur fracture (Fx) and hemorrhagic shock, followed by resuscitation (THFx). Fourteen sham animals received either EtOH or NaCl and underwent surgical procedures without THFx induction. After 24 h, oil red O staining of fatty vacuoles in the liver was performed. Histological lung injury score (LIS) was assessed to analyze the trauma-induced RLI. Gene expression of *Cxcl1*, *Il-1β*, *Muc5ac*, *Tnf*, and *Tnfrsf10b* as well as CXCL1, IL-1β, and TNF protein levels in the lung tissue and bronchoalveolar lavage fluid were determined by RT-qPCR, ELISA, and immunohistological analyses. Infiltrating polymorphonuclear leukocytes (PMNLs) were examined *via* immunostaining. Apoptosis was detected by activated caspase-3 expression in the lung tissue. To confirm active Wnt signaling after trauma, gene expression of *Wnt3a* and its inhibitor sclerostin (*Sost*) was determined. Protein expression of A20 and RIPK4 as possible modulators of the Wnt signaling pathway was analyzed *via* immunofluorescence.

**Results:**

Significant fatty changes in the liver confirmed the acute EI. Histopathology and decreased *Muc5ac* expression revealed an increased lung barrier breakdown and concomitant lung injury after THFx versus sham. EI prior trauma decreased lung injury. THFx increased not only the gene expression of pro-inflammatory markers but also the pulmonary infiltration with PMNL and apoptosis versus sham, while EI prior to THFx reduced those changes significantly. EI increased the THFx-reduced gene expression of *Sost* and reduced the THFx-induced expression of *Wnt3a*. While A20, RIPK4, and membranous β-catenin were significantly reduced after trauma, they were enhanced upon EI.

**Conclusion:**

These findings suggest that acute EI alleviates the uncontrolled inflammatory response and lung barrier breakdown after trauma by suppressing the Wnt/β-catenin signaling pathway.

## Introduction

Traumatic injury is one of the leading causes of worldwide mortality (1). Femur fracture accompanied by hemorrhage is associated with high morbidity and mortality rates (2). Notably, hemorrhagic shock (HS) is characterized by marked early inflammatory response and concomitant activation of immune cells, leading to organ damage and significant mortality (3). Femur fracture in particular can induce remote organ complications, especially in the lungs (4). Lung and remote lung injury (RLI), a common complication after surgical procedures post-trauma, have also been shown in the combinatory model of femur fracture accompanied by hemorrhage (THFx) in mice (5, 6). Many inflammatory pathways and mediators including cytokines and other damage-associated molecular patterns were found to be important triggers for the development of lung injury. Studies have shown that proinflammatory cytokines, among the systemically distributed interleukins (IL)-1β, IL-6, IL-8, and tumor necrosis factor (TNF)-α, were associated with the activation of caspase-3, leading to pulmonary apoptosis and alveolar–capillary barrier dysfunction with the subsequent development of lung injury after trauma (5).

Wnt signaling with its main mediator protein β-catenin is essential in a variety of biological responses, including bone formation and remodeling, organ development and repair, as well as embryogenesis and carcinogenesis (7, 8). The hallmark of the canonical Wnt pathway is to control the β-catenin dynamics and, thus, the activation of β-catenin-mediated transcriptional activity. The cytoplasmic level of β-catenin is tightly regulated *via* its phosphorylation by the “destruction complex”, consisting of the tumor suppressor adenomatous polyposis coli (APC), the scaffold protein AXIN, glycogen synthase kinase (GSK)3β, and casein kinase (CK)1α (7, 9). CKI and GSK3β initiate the phosphorylation of β-catenin, allowing the beta-transducin repeat-containing protein to promote ubiquitination and, thus, the degradation of β-catenin (9). A Wnt member protein (e.g., WNT3A) binding to its receptor inhibits the destruction complex activities, leading to stabilization and accumulation of the cytoplasmic β-catenin, eventually allowing its translocation into the nucleus (7, 9, 10). In the absence of a Wnt stimulus, the majority of β-catenin is located at the cytoplasmic side of the cell membrane in complexes with E-cadherin and α-catenin, preventing β-catenin from degradation (9, 10). β-catenin–E-cadherin-based maintenance and stabilization of adherens junctions promote cell-to-cell adhesion (10). Tumor necrosis factor, alpha-induced protein 3 (TNFAIP3, also known as A20) is indispensable for regulating vascular E-cadherin expression in adherens junctions to maintain and repair damaged endothelial functions after pulmonary vascular injury by lipopolysaccharide (11). Interestingly, there is also an evidence of A20 presumed involvement to Wnt signaling *via* the receptor interacting protein kinase (RIPK)4, which is a positive modulator of the Wnt pathway (12). Over the past decades, significant progress has been made in therapeutics targeting the Wnt/β-catenin signaling pathway in bone regeneration settings, focusing on agents that neutralize or inhibit the negative regulators of Wnt signaling, such as sclerostin, among others (13).

Approximately one-third of trauma-related deaths are accompanied by a positive blood alcohol concentration (BAC), and up to 50% of hospital admissions after trauma involve alcohol/ethanol intoxication (EI) (14, 15). Studies have shown the beneficial effects of an acute EI in experimental trauma *via* reduced systemic and local inflammation, while current data indicate that EI might lead to a higher susceptibility to infections (e.g., sepsis and pneumonia) (3, 16). The overall impact of an acute EI on the trauma/hemorrhage-induced Wnt/β-catenin signaling pathway and subsequent lung barrier breakdown as well as organ damage is unknown. Interestingly, a previous study has shown that alcohol exposure had a negative impact on fracture-associated Wnt signaling required for normal bone fracture repair. Here, the phosphorylation of β-catenin was promoted, targeting the protein for degradation and reducing total β-catenin in the fracture callus (17).

Based on this background, the goal of this study was to investigate the following: (1) the role of the canonical Wnt signaling pathway in the trauma-induced lung injury, and (2) the effects of an acute EI on the lung inflammation and barrier breakdown after THFx. We hypothesize that an acute EI would alleviate the uncontrolled inflammatory response and lung barrier breakdown after trauma by suppressing the Wnt/β-catenin signaling pathway.

## Materials and Methods

### Animal Husbandry

This study was authorized by the local institutional animal care and research advisory committee and permitted by the local government of Lower Saxony, Germany (approval number: 33.12-42502-04-17/2491). Male C57BL/6J mice (17–26 weeks, Janvier Labs, Le Genest-Saint-Isle, France) were used for the experiments and were handled only by persons who had a certificate of the Federation of European Laboratory Animal Science Associations. The animals were kept under standardized conditions in the Central Animal Laboratory of the Hannover Medical School and housed in individual cages with an area of 530 cm^2^ (type IIL cage). Standard softwood granules (Altromin GmbH, Lage, Germany) for laboratory animals were used as litter material. Once per week, the cages, bedding, and the drinking water bottles were changed.

### Group Allocation

Animals were randomly assigned to one of four groups. Mice were gavaged with either sodium chloride (NaCl, ctrl) or ethanol (EtOH) as described in the experimental model. Animals in sham groups (sham ctrl: *n* = 7; sham EtOH: *n* = 7) received an external fixator and catheterization of the femoral artery, but no osteotomy or blood loss was induced. Mice in trauma groups (THFx ctrl: *n* = 7, THFx EtOH: *n* = 7) received the external fixator followed by osteotomy of the femur and HS was induced and maintained by blood withdrawal *via* the catheterized femoral artery.

### Experimental Model

Depending on group assignment, mice received an intragastric gavage of either 0.9% sodium chloride (NaCl, ctrl, 12.66 µl/g body weight) or 35% ethanol (EtOH, >99.5% ethanol diluted in 0.9% sodium chloride, 12.66 µl/g body weight resulting in a 3.5 g EtOH/kg body weight) *via* a rigid blunt cannula 2 h before surgery. An alcohol concentration of 35% was selected to simulate acute EI prior to subsequent procedures. In previous studies, such acute EI generated fatty changes in the liver that confirmed the induced EI (18). In total, the animals received two gavages. In the first gavage, 50% of the total volume (NaCl or EtOH) was administered. After 15 min, the remaining volume was administered *via* the second gavage. The animals were observed for activity, general health condition, posture, signs of pain, and lameness. To achieve a high blood alcohol level in the mice, surgery was started 2 h after gavage. We measured the alcohol concentration in the blood in the Clinical Chemistry Department of the MHH in six mice. They had an alcohol concentration 1.54‰ at 2 h after intoxication. This time point was chosen according to a previous study (19).

All surgical procedures were performed under deep inhalation anesthesia using isoflurane (Baxter Deutschland GmbH, Unterschleißheim, Germany) as described before (20, 21). Interdigital reflexes were proofed regularly and the surgery began when this reflex was negative. During the surgical procedure, mice were warmed using heating pads. An intraoperative analgesia with 5 mg/kg body weight carprofen (Rimadyl^®^, Zoetis Deutschland GmbH, Berlin, Germany) and 1 mg/kg body weight butorphanol (Torbugesic^®^, Zoetis Deutschland GmbH, Berlin, Germany) was injected subcutaneously. Local anesthesia with prilocaine hydrochloride was applied into the sites of the operation. For postoperative analgesia, 200 mg/kg body weight metamizole was added to the drinking water. After the surgery, the animals were kept warm and under red light until full awakening and housed individually in cages to avoid aggressive behavior between male mice and littermates reopening the surgical wound. Postoperative controls of the animals were conducted regularly, and the general health status of the animals as well as activity and possible lameness were controlled.

All surgical procedures were conducted as described before (20, 21). The placement of an external fixator (MouseExFix simple L 100%, RISystem, Davos, Switzerland) to the right femoral bone was performed in the sham and trauma group. In brief, the fixator was implanted according to the manufactuter’s manual and afterwards a diaphyseal osteotomy was performed centrally between the two middle pins using a wire saw with a diameter of 0.44 mm (Gigli wire saw, RISystem). Sham animals (sham ctrl; sham EtOH) only received the external fixator, but no osteotomy was performed. Animals in the trauma groups (THFx ctrl; THFx EtOH) underwent a subsequent pressure-controlled HS. Briefly, a catheter was installed into the femoral artery and blood was collected until a mean arterial blood pressure with a target value of 35 ± 5 mmHg was reached. The shock state of hypovolemia was maintained for 90 min in total. After 90 min, four times the amount of the withdrawn blood (maximum 2.4 ml) was reperfused with body-warm Ringer’s solution over a period of 30 min and the catheter was removed after reinfusion. In the sham animals, only catheterization was performed, but no blood loss was induced. Wounds were closed by suturing with Prolene 6-0 (Ethicon, Cincinnati, USA) and the animals were allowed to move freely immediately after the experimental procedure.

### Harvesting Procedures

Twenty-four hours after surgery, the animals were sacrificed. Mice were anesthetized with 75 mg/kg body weight ketamine and 1 mg/kg body weight medetomidine *via* intraperitoneal injection. After confirming a negative interdigital and tail pinch reflexes, the sacrifice was initiated. The heart was punctured with a heparinized sharp 25-gauge syringe (BD, Franklin Lakes, USA) and followed by cardiac exsanguination for blood collection. Then, mice were killed by cervical dislocation. Abdominal cavity was opened and the incision was extended over the chest wall until the trachea was visible. The trachea was first punctured with a 25-gauge needle (BD, Franklin Lakes, USA) and then a 19-gauge syringe (BD, Franklin Lakes, USA) containing 1.1 ml of phosphate-buffered saline (PBS) was inserted. Lungs were flushed with 1.1 ml of PBS, followed by collection of at least 800 µl of bronchoalveolar lavage fluid (BALF). The BALF was centrifuged at 1,164 × *g* for 5 min at 4°C and the cell-free supernatant was frozen at −80°C for subsequent analyses. The hole in the trachea was closed with a blunt clamp, and perfusion of the entire animal with 20 ml of PBS *via* a 21-gauge blunt syringe (BD, Franklin Lakes, USA) through the heart (apex) began. The left lung was ligated, removed, snap frozen using liquid nitrogen, and stored at −80°C for later analyses. Mice were subsequently perfused with 10 ml of 4% buffered Zn-Formalin (Thermo Fisher Scientific, Waltham, USA) *via* the heart with a 21-gauge syringe (BD, Franklin Lakes, USA). The right liver lobe and right lung were removed for overnight fixation and further histo(morpho)logical analyses.

### Examination of EtOH-Induced Hepatic Fat Accumulation

The evaluation of the fatty vacuoles (oil red O staining) in the liver after 24 h post-trauma confirms an acute EI, which was established and proofed in previous studies (3). Hepatic sections (3 µm) were fixed with 10% buffered Zn-formalin. Lipids were stained for 50–60 min with an oil red O working solution (0.35 g oil red O dissolved in 25 ml of 100% methanol mixed with 10 ml of 1 M NaOH) and counterstained with hematoxylin (5 g/L) for 10 min. The images were captured by using the Zeiss Axio Observer Z1 microscope (40× objective, Zeiss, Göttingen, Germany).

### Examination of Lung Injury

Specimens were fixated in 4% buffered Zn-formalin overnight and embedded in paraffin. For subsequent staining with hematoxylin-eosin (HE), sectioning of 3-µm samples was performed. Lung sections were deparaffinized, rehydrated, and stained with hemalum solution according to Mayer (Carl Roth, Karlsruhe, Germany) for 10 min. After blue annealing in rinsing water (10 min), the tissue was counterstained with eosin (Carl Roth, Karlsruhe, Germany) for 3 min. This was followed by differentiation, dehydration using an ascending alcohol series, and xylene-based mounting medium (Mountex, Medite Medical GmbH, Burgdorf, Germany). An independent examiner determined the histological tissue damage of HE-stained sections of the various experimental groups in a blinded manner. To quantify the histopathological lung injury, sections of lungs were examined for desquamation, dystelectasis/atelectasis, emphysema, congestion, interstitial thickness/infiltration with inflammatory cells, and bronchial exudate by an independent examiner. The orientation for scoring is provided by Matute-Bello et al. (22). The findings from the sham ctrl group were used for normalization as 100%.

### Quantification of Protein Expression Levels in BALF

To assess the extent of lung damage and loss of pulmonary barrier integrity, levels of proinflammatory mediators in the BALF were determined. Lungs of the anesthetized mice were subjected to BAL, and the BALF was processed as described above. Supernatants were stored at −80°C for later analyses of the protein concentration of CXCL1, IL-1β, and IFN-gamma using mouse-specific ELISA kits (R&D Systems, Minneapolis, USA) according to the manufacturer’s instructions. ELISA was performed using the Infinite M200 microplate reader (Tecan, Männedorf, Switzerland).

### Ribonucleic Acid Isolation and Reverse Transcription-Quantitative Polymerase Chain Reaction Analysis

The tissue was homogenized using the Precellys 24 Homogenizer (Bertin Technologies, Montigny-le-Bretonneux, France) according to the manufacturer’s instructions. RNA was then isolated from the homogenate using the RNeasy assay (Qiagen, Hilden, Germany) following the manufacturer’s protocol. To remove residual DNA, the RNase-free DNase kit (Qiagen, Hilden, Germany) was used. The quantity and quality of RNA were determined using the Spark M10 Microplate Reader with the Tecan’s NanoQuant Plate (Tecan, Männedorf, Switzerland). The iScript™ cDNA Synthesis Kit (BioRad, Hercules, USA) was used according to the manufacturer’s instructions for cDNA synthesis. Gene expressions of *Cxcl1* (qMnuCED0047655), *Il-1β* (qMnuCED0045755), *Tnf* (qMnuCED0004141), tnfrsf10b (qMnuCED0046132), *Muc5ac* (qMnuCED0061472), *Wnt3a* (qMnuCID0005162), and *Sost* (qMnuCED0045167) were quantified by using a specific primer for mouse and *Gapdh* (qMnuCED0027467, all PrimePCR SYBR Green Assay, BioRad, Hercules, USA) as housekeeping gene (control). The total volume of the PCR reaction was 25 µl with SYBR green qPCR Master Mix (BioRad) according to the manufacturer’s instructions. The PCR reaction was performed on a C1000 Touch Thermal Cycler with the CFX96 Touch Real-Time PCR Detection System (BioRad, Hercules, USA) and consists of an initial denaturation step at 95°C for 10 min, followed by 40 cycles of denaturation at 95°C for 15 s, and annealing/extension at 60°C for 60 s. The relative gene expression of each target gene normalized to *GAPDH* was calculated by using the 2^−ΔΔCT^ method [comparative threshold-cycle (CT) method].

### Immunohistology Staining of CXCL1, Neutrophile Elastase, and Active Caspase-3

Lung tissue sections (3 µm) were deparaffinized using Roti Histol (Carl Roth, Karlsruhe, Germany) and rehydrated using a descending alcohol series. Heat-induced epitope retrieval (HIER) was performed by using R-Universal epitope recovery buffer (Aptum, Kassel, Germany) in the 2100-Retriever (Prestige Medical, Blackburn, England) for 60 min at 121°C. Blocking of endogenous peroxidase was performed *via* hydrogen peroxide (Peroxidase UltraVision Block, Thermo Fisher Scientific, Waltham, USA) for 20 min. Primary antibodies for CXCL1 (abcam, USA, rabbit anti-mouse, 1:300), neutrophil elastase (Bioss, USA, rabbit anti-mouse, 1:200), and active caspase-3 [Cell Signaling Technology, USA, anti-cleaved caspase-3 (Asp175), #9661, rabbit anti-mouse] were diluted as suggested by the manufacturer in Antibody Dilution Buffer (Dako Cytomation) and incubated for 1 h at room temperature. Subsequently, the secondary antibody conjugated with horseradish peroxidase (HRP) [Histofine Simple Stain Mouse MAX PO (R), Nichirei Biosciences Inc.] was applied for 30 min at room temperature and 3-amino-9-ethylcarbazol (AEC, DCS Innovative Diagnostik-Systeme, Hamburg) was used to detect specific binding. Slides were counterstained with hematoxylin (Carl Roth, Karlsruhe, Germany) and mounted (Medite Medical GmbH, Burgdorf, Germany). Imaging was performed using the Zeiss Axio Observer Z1 microscope (40× objective, Zeiss, Göttingen, Germany). The evaluation was done *via* ImageJ software. For CXCL1, mean intensity values were measured for all groups, whereas for neutrophil elastase and active caspase-3, positive-counted cells were measured in 25 high-power fields (HPF) at 400× magnification.

### Immunofluorescent Staining of A20, β-Catenin, E-Cadherin, RIPK4, and CD4 T Cells

Paraffin-embedded lung tissue sections (3 µm) were deparaffinized with Roti Histol (Carl Roth, Karlsruhe, Germany) (2× 5 min) and rehydrated with a descending alcohol series. HIER was performed using R-Universal Epitope Recovery Buffer (Aptum, Kassel, Germany) for 10 min at 90°C in a preheated water bath. Slides were then cooled to room temperature in distilled water for 30 min and washed in distilled water (3× 5 min) and DPBS (Thermo Fisher Scientific, USA) (1× 5 min). Permeabilization was performed with 0.1% Tween 20 (Merck, Darmstadt, Germany) in DPBS (Thermo Fisher Scientific, USA) for 10 min at RT followed by washing in DPBS (1× 5 min and 2× 3 min) (Thermo Fisher Scientific, USA). Permeabilization was followed by blocking the slides with BSA (1%, Merck, Darmstadt, Germany) in PBST (PBS with 0.1% Tween 20) for 30 min at RT in a humidified incubation chamber. After blocking, slides were washed again in distilled water (1× 5 min) and DPBS (1× 5 min and 2× 3 min) (Thermo Fisher Scientific, USA). Lung sections were incubated with primary antibodies against A20 (abcam, USA, rabbit anti-mouse, 1:500, cat. ab92324), β-catenin (antibodies-online GmbH, Aachen, Germany, rabbit anti-mouse, 1:500, cat. ABIN2855042), CD4 (BD, rat anti-mouse, 1:50, cat. 550278), E-cadherin (Purified mouse anti-E-cadherin, BD Biosciences, 1:500, 610182), and RIPK4 [Santa Cruz, USA, mouse anti-mouse, RIP4 Antibody (E-7) Alexa Fluor^®^ 647, 1:200, cat. sc-377368 AF647] overnight at 4°C in a humidified incubation chamber, which were diluted in blocking buffer (1% BSA in PBST) according to the manufacturer’s instructions. Incubation of primary antibodies was followed by washing according to the washing steps after blocking. The next steps were performed in the dark. The secondary antibodies (Thermo Fisher Scientific, USA Alexa Fluor^®^ 488, donkey anti-rabbit, Alexa Fluor 568 donkey anti-Mouse IgG, and goat anti-rat Alexa Fluor^®^ 647, 1:1,000) were diluted in blocking buffer according to the manufacturer’s instructions and applied to the A20 and β-catenin slides, where it was stained for 60 min at RT in a humidified incubation chamber. This was followed by another wash in DPBS (3× 5 min) (Thermo Fisher Scientific, USA). RIPK4 primary antibody was conjugated with Alexa Fluor^®^ 647 (Molecular Probes, Inc., Oregon, USA). Tissue slides were counterstained with DAPI (Cambrex Bioscience, USA, 1:10,000) for 10 min with subsequent washing in DPBS (4× 5 min) (Thermo Fisher Scientific, USA) and mounted using Gold Antifade Mountant (Invitrogen™-ProLong™). The slides were sealed with nail polish after mounting. Imaging was performed using the Zeiss Axio Observer Z1 microscope (40× objective, Zeiss, Göttingen, Germany). Pictures at 400× magnification were evaluated with the FIJI (ImageJ) software. Mean intensity values were measured, and means were calculated for each group. Colocalization analysis of β-catenin–E-cadherin complexes was performed on fluorescent sections using ZEN Pro-software 3.2. The images for analysis were obtained using a ZEISS Axio Observer fluorescent microscope and the Zeiss digital camera ZEISS Axiocam (705 mono) with ×63 objective. Each analyzed image was individually evaluated for validation of the pattern of the staining used before processing. The images were opened in the ZEN Pro colocalization module, and the region of interest (ROI) for colocalization was defined by allowing fluorescence channels to concentrate on the relevant parts of the image. A colocalized channel was created; and channel statistics were calculated.

### Statistical Analysis

Statistical analysis was performed using GraphPad Prism 6 (GraphPad Software, Inc., San Diego, CA). Based on the histogram and Shapiro–Wilk test, the non-parametric Kruskal–Wallis test, which does not assume a normal distribution of the residuals, followed by Dunn’s *post hoc* test for the correction of multiple comparisons was applied. Results were expressed as mean and standard error of the mean. A *p*-value of less than 0.05 was considered to be statistically significant.

## Results

### Impact of Acute EI on Lung Damage After THFx

**Figure 1:**
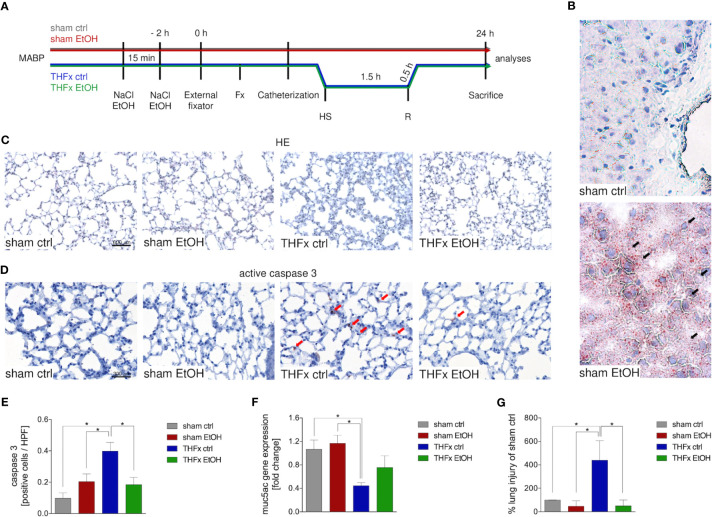
Impact of acute ethanol intoxication (EI) on lung damage after femoral fracture (Fx) and hemorrhagic shock (HS). **(A)** Experimental design is shown. Two hours before the initiation of experiments, the animals received an intragastric gavage of either sodium chloride (NaCl, ctrl, *n* = 14) or ethanol (EtOH, *n* = 14) to simulate an acute intoxication with alcohol. Trauma groups underwent Fx (*via* osteotomy after the placement of an external fixator) and a pressure-controlled HS with subsequent resuscitation (R) with Ringer’s solution (TH) *via* catheterized femoral artery (*n* = 14). Sham groups underwent catheterization and received an external fixator, but THFx was not induced (*n* = 14). Twenty-four hours after the end of the experiment, mice were euthanized, and sampling was performed. MABP: mean arterial blood pressure. **(B)** Representative oil red O staining of fatty vacuoles in hepatic tissue of mice undergoing sham procedure. Fatty vacuoles are colored red (black arrow). **(C)** Representative lung sections upon hematoxylin/eosin (HE) staining of the sham ctrl, sham EtOH, THFx ctrl, and THFx EtOH groups are shown. **(D)** Representative lung sections upon the staining of activated caspase-3 in the sham ctrl, sham EtOH, THFx ctrl, and THFx EtOH groups are shown. Exemplary caspase-3-positively stained cells are marked with red arrows. **(E)** Quantification of caspase 3-positively stained cells per high-power field (HPF) is shown. **(F)** RT-qPCR with homogenized lung tissue was performed. Relative gene expression of Muc5ac normalized to Gapdh in lung tissue was calculated by using the comparative threshold-cycle 2^−ΔΔCT^ method. **(G)** Quantification of the histopathological lung injury. The findings from the sham ctrl group were used for normalization as 100%. **(E, F)** Data are presented as mean ± standard error of the mean; *n* = 7 in all groups, *p < 0.05 between indicated groups.

Animals received a gavage of either NaCl or EtOH 2 h before the procedures as shown in **Figure 1A**. Mice receiving EtOH showed a clear accumulation of fatty vacuoles in liver 24 h after the procedure compared to animals receiving sodium chloride, confirming successfully induced acute EI as displayed in **Figure 1B**.

Lung organ damage caused by systemic inflammation following THFx was measured by assessing the relative lung injury and compared to the sham ctrl. The histomorphological differences between the groups in the lung after HE staining are demonstrated in **Figure 1C**. A significantly increased histopathological lung injury, e.g., thickening of the alveolar walls/loss of alveolar space, is found in the THFx ctrl group vs. all other groups (*p* < 0.05, **Figures 1C, G**). THFx EtOH exerts a significantly lower relative lung injury vs. THFx ctrl (*p* < 0.05).

The association of the proinflammatory changes with apoptosis induction is shown in **Figure 1D** as representative staining of active caspase-3 (red arrows). Quantification of cells that are positive for active caspase-3 as a direct indicator of apoptosis indicates significantly increased apoptosis in the THFx ctrl group vs. all groups (*p* < 0.05, **Figure 1E**). However, acute EI in the THFx significantly decreased caspase-3-positive cells vs. THFx ctrl (*p* < 0.05).

Furthermore, the gene expression of *Muc5ac* coding for its lung protective protein in homogenized lung tissue was investigated (23). Both sham groups had significantly higher relative gene expression compared to THFx ctrl (*p* < 0.05, **Figure 1F**). THFx EtOH does not significantly differ from the sham groups. There was a tendency to upregulate *Muc5ac* gene expression in the THFx EtOH group vs. THFx ctrl (**Figure 1F**).

### Impact of Acute EI on CXCL1 Expression and Neutrophilic Infiltration After THFx

Both protein and gene expression of CXCL1 in homogenized lung tissue and CXCL1 protein concentration in the BALF were assessed.

**Figure 2:**
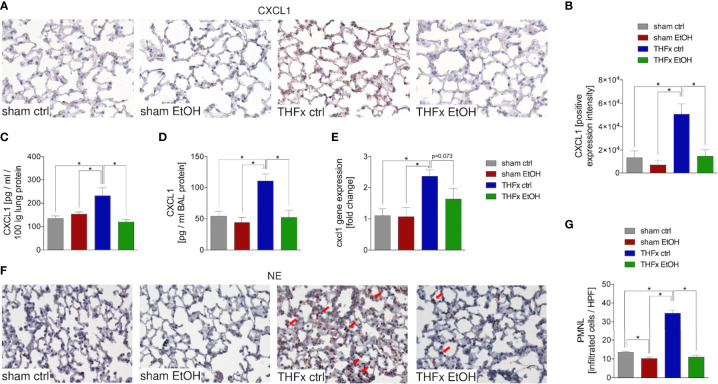
Impact of acute ethanol intoxication (EI) on Cxcl1 gene and CXCL1 protein expression and neutrophilic infiltration after femoral fracture (Fx) and hemorrhagic shock (HS). The animals received an intragastric gavage of either sodium chloride (ctrl, *n* = 14) or ethanol (EtOH, *n* = 14) to simulate an acute intoxication with alcohol. Two hours before the initiation of experiments, the animals in trauma groups underwent an Fx (*via* osteotomy after the placement of an external fixator) and a pressure-controlled HS with subsequent resuscitation with Ringer’s solution (THFx) *via* catheterized femoral artery (*n* = 14). Sham groups underwent catheterization and received an external fixator, but THFx was not induced (*n* = 14). Twenty-four hours after the end of the experiment, mice were euthanized, and sampling was performed. **(A)** Representative lung sections upon the staining of CXCL1 in the sham ctrl, sham EtOH, THFx ctrl, and THFx EtOH groups are shown. **(B)** Quantification of CXCL1-positively stained cells per high-power field (HPF) is shown. **(C)** Quantification of CXCL1 protein concentration in lung tissue homogenates using mouse-specific ELISA kits is shown. **(D)** Quantification of CXCL1 protein concentration in bronchoalveolar lavage (BAL) fluid is represented. Briefly, to obtain BAL, *via* trachea, lungs were flushed with 1.1 ml of phosphate buffered saline and then 800 µl was withdrawn for the determination of total protein concentration. **(E)** RT-qPCR with homogenized lung tissue for Cxcl1 gene expression analyses is depicted. The relative gene expression of Cxcl1 normalized to Gapdh was calculated by using the comparative threshold-cycle 2^−ΔΔCT^ method. **(F)** Representative immune histological staining of neutrophil elastase (NE) as a marker of polymorphonuclear leukocytes (PMNLs) in lung sections is represented. Red arrows indicate NE-positively stained cells. **(G)** Quantification of NE-positively stained cells per high-power field (HPF) is shown. Data are presented as mean ± standard error of the mean; *n* = 7 in all groups, *p < 0.05 between indicated groups.

Representative immunostaining of CXCL1 is shown in **Figure 2A**, and the mean intensity values are demonstrated in **Figure 2B**. Comparison of the mean intensity values between the four groups showed the same pattern as observed regarding the protein expression of CXCL1 in the lungs and CXCL concentration in the BALF. The marked local remote proinflammatory response to trauma is confirmed by the significantly increased CXCL1 expression in the lung in THFx ctrl vs. sham ctrl and also sham EtOH (*p* < 0.05, **Figures 2A, B**). CXCL1 expression in the THFx EtOH group is significantly reduced vs. the THFx ctrl group (*p* < 0.05, **Figure 2B**).

Protein expression of CXCL1 in lung tissue is significantly increased in THFx ctrl vs. all other groups (*p* < 0.05, **Figure 2C**). Trauma animals undergoing EI have significantly decreased protein expression vs. THFx ctrl (*p* < 0.05, **Figure 2C**).

CXCL1 protein expression in the BALF was significantly increased in THFx ctrl vs. all other groups (*p* < 0.05, **Figure 2D**), comparable to results that were observed for CXCL1 protein concentration in the lung tissue homogenates. In THFx EtOH, CXCL1 protein expression is significantly lower vs. THFx ctrl (*p* < 0.05, **Figure 2D**).

Relative gene expression of *Cxcl1* does not significantly differ between sham ctrl and sham EtOH (**Figure 2E**). Significantly higher relative gene expression of *Cxcl1* in THFx ctrl vs. both sham groups is observed (*p* < 0.05, **Figure 2E**). However, there is a trend to decreased relative gene expression in THFx EtOH vs. THFx ctrl (*p* = 0.073, **Figure 2E**).

As CXCL1 plays an important role in attracting neutrophils, we investigated neutrophil recruitment to the lungs (24). Neutrophil infiltration of lung tissue was assessed by immunohistology staining (**Figure 2F**) and quantified by counting the positively stained cells (**Figure 2G**). Neutrophil infiltration is significantly increased in THFx ctrl vs. all other groups (*p* < 0.05). Compared to THFx ctrl, in THFx EtOH, a significantly reduced neutrophil infiltration is shown (*p* < 0.05, **Figure 2G**). In sham EtOH, a significantly lower neutrophil invasion vs. sham ctrl is observed (*p* < 0.05, **Figure 2G**).

### Impact of Acute EI on IL-1β and TNF as Well as Tnfrs10b After THFx

**Figure 3:**
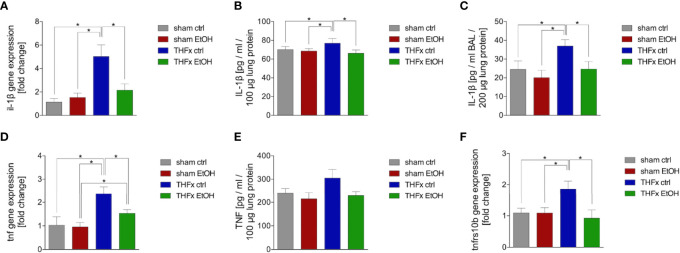
Impact of acute ethanol intoxication (EI) on IL-1β and TNF as well as Tnfrs10b after femoral fracture (Fx) and hemorrhagic shock (HS, THFx respectively). The animals received an intragastric gavage of either sodium chloride (ctrl, *n* = 14) or ethanol (EtOH, *n* = 14) to simulate an acute intoxication with alcohol. Two hours before the initiation of experiments, the animals in trauma groups underwent an Fx (*via* osteotomy after the placement of an external fixator) and a pressure-controlled HS with subsequent resuscitation with Ringer’s solution (THFx) *via* catheterized femoral artery (*n* = 14). Sham groups underwent catheterization and received an external fixator, but THFx was not induced (*n* = 14). Twenty-four hours after the end of the experiment, mice were euthanized, and sampling was performed. **(A)** RT-qPCR of homogenized lung tissue has been performed. The relative gene expression of Il-1β normalized to Gapdh was calculated by using the 2^−ΔΔCT^ method. **(B)** Quantification of IL-1β protein concentration in total lung tissue protein homogenates using mouse-specific ELISA kits is shown. **(C)** Quantification of IL-1β protein concentration in bronchoalveolar lavage fluid (BALF) is represented. Briefly, to obtain BALF, *via* trachea, lungs were flushed with 1.1 ml of phosphate buffered saline and then 800 µl was withdrawn for the determination of total protein concentration and IL-1β levels. **(D)** The relative gene expression of Tnf normalized to Gapdh after RT-qPCR was calculated by using the comparative threshold-cycle 2^−ΔΔCT^ method. **(E)** Quantification of TNF protein concentration in total lung tissue protein homogenates using mouse-specific ELISA kits is shown. **(F)** The relative gene expression of Tnfrs10b normalized to Gapdh was assessed *via* RT-qPCR and calculated by using the comparative threshold-cycle 2^−ΔΔCT^ method. Data are presented as mean ± standard error of the mean; *n* = 7 in all groups, *p < 0.05 between indicated groups.

Similar to CXCL1, we investigated Il-1β gene expression and IL-1β protein expression in homogenized lung tissue as well as protein concentration in the BALF. The relative gene expression of Il-1β was significantly higher in THFx ctrl vs. all other groups (*p* < 0.05, **Figure 3A**). Il-1β gene expression is significantly reduced in THFx EtOH vs. THFx ctrl (*p* < 0.05, **Figure 3A**).

IL-1β concentration in the lung tissue protein homogenates was significantly increased in THFx ctrl vs. all other groups (*p* < 0.05, **Figure 3B**). IL-1β concentration was significantly reduced in THFx EtOH vs. THFx ctrl (*p* < 0.05, **Figure 3B**). There are no differences between the sham groups.

IL-1β concentration in the BALF was significantly enhanced in THFx ctrl vs. all other groups (*p* < 0.05, **Figure 3C**). In THFx EtOH, IL-1β levels were significantly reduced vs. THFx ctrl (*p* < 0.05). Sham animals did not differ significantly.

There was a significantly higher relative gene expression of Tnf in THFx ctrl vs. all other groups (*p* < 0.05, **Figure 3D**). Tnf gene expression was significantly decreased in THFx EtOH vs. THFx ctrl (*p* < 0.05, **Figure 3D**). However, Tnf gene expression in THFx ETOH was significantly increased vs. sham EtOH (*p* < 0.05, **Figure 3D**).

Compared with the significant differences that are observed regarding the *Tnf* gene expression, only a tendency regarding the TNF protein expression in lung tissue among the respective groups was observed (**Figure 3E**). Between all groups, the highest protein expression was shown in THFx ctrl.

The relative gene expression of *Tnfrsf10b* did not differ between both sham groups (**Figure 3F**). In THFx ctrl, a significantly increased relative *Tnfrsf10b* gene expression vs. all other groups was detected (*p* < 0.05, **Figure 3F**). A significantly decreased relative gene expression of Tnfrsf10b in THFx EtOH vs. THFx ctrl was shown (*p* < 0.05, **Figure 3F**).

### Impact of Acute EI on Wnt Signaling, β-Catenin–E-Cadherin Co-Expression, and A20 After THFx

**Figure 4:**
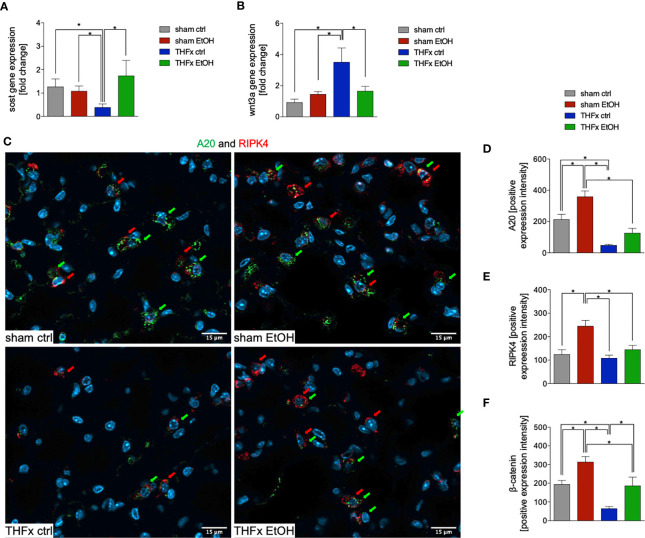
Impact of acute ethanol intoxication (EI) on Wnt signaling and A20 after femoral fracture (Fx) and hemorrhagic shock (HS, THFx respectively). The animals received an intragastric gavage of either sodium chloride (ctrl, *n* = 14) or ethanol (EtOH, *n* = 14) to simulate an acute intoxication with alcohol. Two hours before the initiation of experiments, the animals in trauma groups underwent an Fx (*via* osteotomy after the placement of an external fixator) and a pressure-controlled HS with subsequent resuscitation with Ringer’s solution (THFx) *via* catheterized femoral artery (*n* = 14). Sham groups underwent catheterization and received an external fixator, but THFx was not induced (*n* = 14). Twenty-four hours after the end of the experiment, mice were euthanized, and sampling was performed. Wnt3a and its inhibitor sclerostin (Sost) were determined. **(A)** RT-qPCR of homogenized lung tissue was performed. The relative gene expression of Sost, an Wnt inhibitor, normalized to Gapdh was calculated by using the comparative threshold-cycle 2^−ΔΔCT^ method. **(B)** RT-qPCR of Wnt3a is shown after Wnt3a relative gene expression normalization to Gapdh by using the comparative threshold-cycle 2^−ΔΔCT^ method. **(C)** Representative immunofluorescent staining of A20 in green (green arrows, Alexa Fluor 488) and receptor interacting protein kinase 4 in red (RIPK4, red arrows, Alexa Fluor 647) are represented. **(D)** Quantification of A20-positively stained cells per high-power field (HPF) is shown. **(E)** Quantification of RIPK-4 positively stained cells per HPF is shown. **(F)** Immunofluorescence staining of the β-catenin as the key mediator of the Wnt/β-catenin signaling pathway in lung sections of mice was performed, and quantification of the positively stained area per HPF is depicted. Data are presented as mean ± standard error of the mean; *n* = 7 in all groups, *p < 0.05 between indicated groups.

The relative gene expression of *Sost* was examined. The THFx ctrl group had a significantly reduced *Sost* gene expression vs. all other groups (*p* < 0.05, **Figure 4A**). In THFx EtOH, a significantly increased *Sost* gene expression vs. THFx ctrl was detected (*p* < 0.05, **Figure 4A**). Sham groups did not differ significantly.

Among all groups, the highest and most significant increase in relative *Wnt3a* gene expression in THFx ctrl vs. all other groups was detected (*p* < 0.05, **Figure 4B**). Relative *Wnt3a* gene expression was significantly reduced in THFx EtOH vs. THFx ctrl (*p* < 0.05, **Figure 4B**).

The protein expression of A20 was investigated *via* quantification of the mean intensity values in IF. Representative pictures are demonstrated in **Figure 4C**. A20 expression was significantly reduced in THFx ctrl vs. both sham groups (*p* < 0.05, **Figure 4D**). A20 expression in THFx EtOH did not statistically differ from sham ctrl. However, A20 was significantly reduced in THFx EtOH vs. sham EtOH (*p* < 0.05, **Figure 4D**). Sham EtOH showed a significantly higher A20 expression vs. the sham ctrl group (*p* < 0.05, **Figure 4D**). This increase upon EI was also observed after THFx; however, this tendency was not significant vs. THFx ctrl (**Figure 4D**).

We also examined the association of A20 to RIPK4 in lung tissue by IF, which is representatively shown in **Figure 4C** and quantified in **Figure 4E**. RIPK4 expression in lung tissue showed a significant increase in sham EtOH vs. all other groups (*p* < 0.05, **Figure 4E**). No significant differences among the other groups were found. RIPK4 expression increased in THFx EtOH compared with THFx ctrl; however, this tendency was not significant (**Figure 4E**).

In IF, the expression and distribution of β-catenin protein was determined and compared to the mean intensity values among the groups. The β-catenin levels were significantly enhanced in sham EtOH vs. sham ctrl and against both trauma groups (*p* < 0.05, **Figure 4F**). The expression levels of β-catenin were significantly decreased in THFx ctrl vs. all other groups (*p* < 0.05, **Figure 4F**). EI prior to trauma significantly increased the β-catenin levels vs. THFx ctrl (*p* < 0.05, **Figure 4F**). In both trauma groups, the β-catenin expression was significantly reduced compared to the corresponding control groups, respectively (*p* < 0.05, **Figure 4F**).

**Figure 5:**
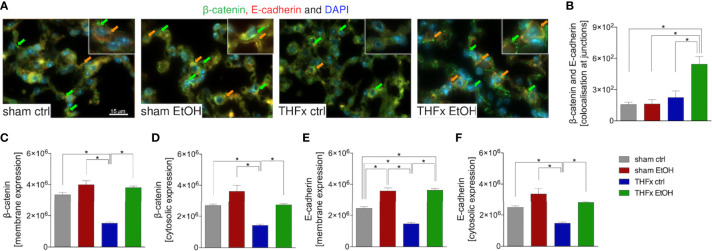
Impact of acute ethanol intoxication (EI) on the β-catenin–E-cadherin co-expression after femoral fracture (Fx) and hemorrhagic shock (HS, THFx respectively). The animals received an intragastric gavage of either sodium chloride (ctrl, *n* = 14) or ethanol (EtOH, *n* = 14) to simulate an acute intoxication with alcohol. Two hours before the initiation of experiments, the animals in trauma groups underwent an Fx (*via* osteotomy after the placement of an external fixator) and a pressure-controlled HS with subsequent resuscitation with Ringer’s solution (THFx) *via* catheterized femoral artery (*n* = 14). Sham groups underwent catheterization and received an external fixator, but THFx was not induced (*n* = 14). Twenty-four hours after the end of the experiment, mice were euthanized, and sampling was performed. Expression of β-catenin and E-cadherin as well as their co-localization were determined. **(A)** Representative immunofluorescent staining of β-catenin in green (green arrows, Alexa Fluor 488) and E-cadherin in red (Alexa Fluor 568), and overlay (orange arrows) are represented. **(B)** Quantification of the β-catenin–E-cadherin co-expression at junctions is shown. **(C)** Quantification of β-catenin expression at the membrane, **(D)** β-catenin expression in the cytosol, **(E)** E-cadherin at the membrane, and **(F)** E-cadherin in the cytosol in lung sections of mice is depicted. Data are presented as mean ± standard error of the mean; *n* = 7 in all groups, *p < 0.05 between indicated groups.

Stratifying the localization of the β-catenin and E-cadherin expression (**Figure 5A**) has shown that the colocalization of β-catenin–E-cadherin complexes was significantly higher in THFx vs. all other groups (*p* < 0.05, **Figure 5B**). However, expression of β-catenin was significantly increased in all groups vs. the THFx ctrl group, both membranous (*p* < 0.05, **Figure 5C**) and cytosolic expression (*p* < 0.05, **Figure 5D**). Membranous E-cadherin expression was significantly enhanced in both EtOH groups vs. the corresponding ctrl groups (*p* < 0.05, **Figure 5E**), while THFx showed significantly less E-cadherin presence at the membrane vs. all other groups (*p* < 0.05, **Figure 5E**). The significantly reduced E-cadherin presence in THFx vs. all other groups is also observed in the cytosol (*p* < 0.05, **Figure 5F**).

### Impact of Acute EI on CD4 T Cell Infiltration After THFx

**Figure 6:**
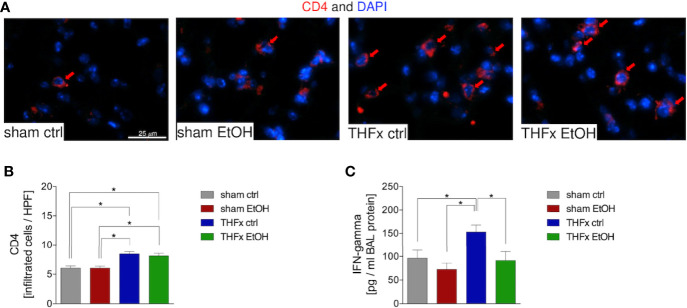
Impact of acute ethanol intoxication (EI) on CD4 T-cell infiltration after femoral fracture (Fx) and hemorrhagic shock (HS, THFx respectively). The animals received an intragastric gavage of either sodium chloride (ctrl, *n* = 14) or ethanol (EtOH, *n* = 14) to simulate an acute intoxication with alcohol. Two hours before the initiation of experiments, the animals in trauma groups underwent an Fx (*via* osteotomy after the placement of an external fixator) and a pressure-controlled HS with subsequent resuscitation with Ringer’s solution (THFx) *via* catheterized femoral artery (*n* = 14). Sham groups underwent catheterization and received an external fixator, but THFx was not induced (*n* = 14). Twenty-four hours after the end of the experiment, mice were euthanized, and sampling was performed. **(A)** Immunofluorescence staining of CD4 in red (red arrows, Alexa Fluor 647) in lung sections of mice was performed, and **(B)** quantification of the positively stained cells per HPF is depicted. **(C)** Quantification of interferon (IFN)-gamma protein concentration in bronchoalveolar lavage (BAL) fluid is represented. Data are presented as mean ± standard error of the mean; *n* = 7 in all groups, *p < 0.05 between indicated groups.

Representative immunostaining of CD4 is shown in **Figure 6A**, and the quantification of counted CD4-positive cells is shown in **Figure 6B**. The marked local proinflammatory response to trauma is confirmed by the significantly increased CD4 T cell infiltration in the lung in THFx ctrl vs. sham ctrl and also sham EtOH (*p* < 0.05, **Figures 6A, B**). Infiltration with CD4-positive cells in the THFx EtOH group is also significantly increased vs. sham ctrl as well as vs. sham EtOH (*p* < 0.05, **Figures 6A, B**).

IFN-gamma protein expression in the BALF was significantly increased in THFx ctrl vs. all other groups (*p* < 0.05, **Figure 6C**), comparable to results that were observed for CXCL1 gene and protein expression.

**Figure 7:**
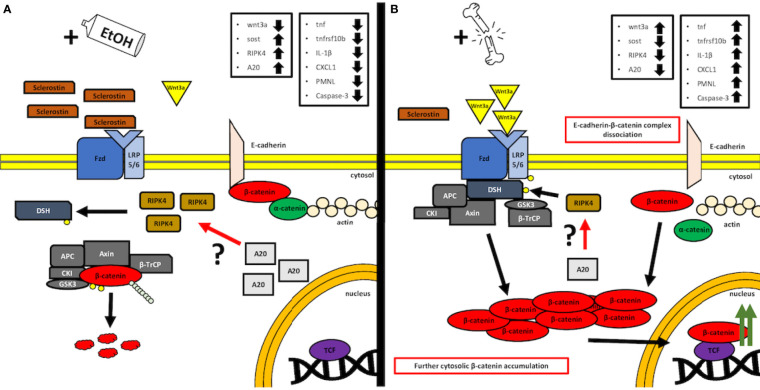
A simplified, graphical depiction of the regulation mechanism of the Wnt/β-catenin signaling by the acute ethanol intoxication (EI) **(A)** and with the major trauma injury **(B)**. **(A)** Suspected mechanism of the EI influence over the activity of the canonical Wnt/β-catenin. According to our findings, we observed that acute alcohol intake leads to the elevated levels of Sost gene expression, whose product, sclerostin, acts as a natural competitor inhibitor for Wnt receptors. Additionally, we noticed increased expression levels of both receptor-interacting protein kinase 4 (RIPK4) and A20 protein in the same EI setting. Interestingly, it was reported before that RIPK4 appears to be a positive regulator of canonical Wnt/β-catenin signaling, while A20 protein seems to be the natural inhibitor of RIPK4 activity and presumably regulates the Wnt/β-catenin signaling through this kinase (12). In our findings, the upregulated levels appear to be contradictory to these results; however, both levels of A20 and RIPK4 seem to compensate each other, and thus, one may suspect an interaction between those, which cancels the RIPK4-upregulating activity, as the Wnt/β-catenin-driven trauma-induced inflammation is suppressed compared to the ctrl group. However, further investigation is required, especially on the field of the base protein expression and gene transcription and the involvement of the alcohol as a potential regulator of A20–RIPK4 interplay. **(B)** Trauma-based breakdown of the lung barrier through the positive regulation of Wnt/β-catenin signaling in the presence of alcohol. The major trauma injury affects the internal sclerostin and wnt3a ligand competition of the Fzd and LRP-5/6 receptor by favoring the expression of the latter, thus stimulating the destruction complex disruption and β-catenin release to the cytosol. In addition, β-catenin appears to dissociate from E-cadherin-based AJ (adherens junction) complexes, thus disrupting the cell–cell interactions of epithelial cells. At the same time, the expression levels of A20 and RIPK4 seem to decrease; however, it is unknown if this downregulation influences the withdrawal of the destruction complex from the cytosol or maybe it is a time-dependent interaction that requires kinetics-based observations, so that the fold change in time of both molecules during the transition between off and on states can be followed. APC: adenomatous polyposis coli, AXIN: the axis inhibition protein scaffold protein, βTrCP: the beta-transducin repeat containing protein, CKI: casein kinase, Fzd: frizzled protein, GSK3β: glycogen synthase kinase 3β, LRP 5/6: low-density lipoprotein receptor-related protein 5/6.

The proposed, presumed mechanism of action is demonstrated in **Figure 7**.

## Discussion

In this work, we aimed to analyze the basic mechanisms of lung barrier disruption and organ damage after THFx with and without EI. Acute alcohol abuse resulting in EI has been implicated as a major risk factor not only for traumatic injury itself but also for post-traumatic immune regulation and recovery. The results thus far have been equivocal. This is of particular importance, as it is estimated that up to 50% of trauma patients present acutely intoxicated (25, 26). Thus, there is a significant need for understanding the influence of acute EI on the inflammatory response and lung injury after trauma. In particular, this study examined the involvement of the canonical Wnt/β-catenin signaling pathway after trauma.

Our results demonstrate that THFx triggered lung injury in mice, which developed from an uncontrolled inflammatory response, e.g., increased expression of TNF-α, IL-1β, CXCL1, and lung infiltration with PMNL, as well as apoptosis, which promotes lung injury. The current investigation explored whether the canonical Wnt/β-catenin signaling pathway, which plays a key role in repair mechanisms and cell-to-cell adhesion, will contribute to the breakdown of the lung barrier after trauma (27, 28). Our findings demonstrate THFx-induced activation of the canonical Wnt *via* inhibition of the destruction complex, since expression of *Wnt3a* increased and that of *Sost* decreased, altogether leading to the stabilization and accumulation of the cytoplasmic β-catenin levels, allowing its translocation into the nucleus. Furthermore, reduced levels of β-catenin, which ultimately lead to the absence of β-catenin in the E-cadherin–β-catenin complexes at the cytoplasmic side of the cell membrane, suggest loss of cell-to-cell adherence and lung barrier breakdown after THFx. In this study, we found that acute EI alleviated the uncontrolled inflammatory response and lung barrier breakdown *via* the canonical Wnt/β-catenin signaling pathway.

Prior studies have shown that severe trauma causes damage to trauma-remote organs such as lungs or liver, mainly by an uncontrolled systemic and local proinflammatory response (4–6). Zhang et al. found increased inflammation and lung damage as also shown by the LIS after hip fracture in rats (29). These results are in line with our findings. We have shown that reduction of the lung-protective *Muc5ac* (23) is associated with enhanced lung injury after THFx. It is well known that mediators of inflammation correlate with the magnitude of the injury and subsequent organ failure in trauma (30). Zhang et al. found increased circulating levels of TNF-α and IL-1β 24 h after traumatic fracture and surgery (29), while Xu et al. also demonstrated increased IL-1β levels in the BALF after HS in mice (31). Here, we demonstrate that lung injury is associated with significantly increased local TNF-α and IL-1β in lungs after THFx. Furthermore, we found THFx-induced *Cxcl1* gene as well as CXCL1 protein expression in the lungs and BALF. As CXCL1 is an important neutrophil chemoattractant (32), the observed increase in PMNL lung infiltration after THFx was expected. These observations are supported by other experimental trauma studies, showing increased CXCL1 and infiltration of inflammatory cells into lungs after experimental multiple trauma models/polytrauma (6, 33). An uncontrolled inflammatory response with excessive cytokine production and leukocyte recruitment can lead to cellular apoptosis/necrosis, tissue damage, hemodynamic changes, and organ failure (34–36). However, the cell counts of neutrophils in the BALF have not been performed. Given the excessive inflammatory mediator release and lung injury after THFx, apoptosis in which caspase activation plays a central role was analyzed. In agreement with Zhang et al., who assessed increased levels of active caspase-3 in lung tissue 24 h after traumatic fracture induction (29), we found markedly increased caspase-3 activation after THFx, indicating lung barrier disruption.

Given the fact that increased apoptosis is associated with increased lung barrier disruption and pulmonary permeability (5), and furthermore, Wnt/β-catenin signaling pathway plays a key role in repair mechanisms and cell-to-cell adhesion (27, 28), we examined this pathway. It is well known that Wnt (e.g., WNT3A) binding to its receptor inhibits the destruction complex, leading to accumulation of the cytoplasmic β-catenin, allowing its translocation into the nucleus (7, 9, 10). Furthermore, in the absence of Wnt stimulus, the majority of β-catenin is located at the cytoplasmic side of the cell membrane in adherens junction-stabilizing complexes with E-cadherin and α-catenin, preventing β-catenin from degradation (9, 10). Activation of the Wnt/β-catenin signaling pathway leads to the release of β-catenin from complexes with E-cadherin (7, 9, 37), and thereby might contribute to the breakdown of the lung barrier. β-catenin signaling is tightly regulated in the early phases of fracture healing, and alterations to β-catenin signaling can play a disparate role in fracture healing (38). Although the Wnt/β-catenin signaling pathway plays a pivotal role in fracture healing, its role in remote organ damage after trauma is still largely unknown. Sawant et al. demonstrated caspase-3 activity, microvascular permeability, and an active canonical Wnt signaling pathway in rat lung microvascular endothelial cells after HS (37). Furthermore, specific inhibition of β-catenin phosphorylation and, thus, inhibition of the trauma-induced Wnt/β-catenin signaling protected from caspase-3 enzyme activity and against microvascular hyperpermeability following HS (37). In the current study, THFx-induced expression of *Wnt3a*, a major trigger for the active canonical Wnt/β-catenin signaling pathway (37), and THFx-reduced expression sclerostin, an inhibitor of the Wnt signaling pathway (13), demonstrate that THFx also induces the Wnt/β-catenin signaling pathway. Therefore, we hypothesize that enhanced canonical Wnt/β-catenin signaling pathways and loss of β-catenin from the membranous complexes as confirmed by the IF cause lung barrier breakdown and lung injury after THFx.

In this study, we focused on acute EI to elaborate its effects on the lung inflammation and barrier breakdown after THFx. Therefore, no unambiguous conclusions can be drawn regarding subacute or chronic EI. The current data regarding the impact of an acute EI on the inflammatory response and organ damage after trauma are conflicting (3, 18, 39, 40). In this study, we found that EI reduced lung damage after THFx. In agreement with our previous findings in experimental traumatic brain injury (18), we found that acute EI decreased the proinflammatory mediators that were released and activated after THFx. EI-reduced expression of *Tnf* and *Il-1β* after THFx in this study is supported by Xu et al. demonstrating reduced expressions of *Tnf-α* and *Il-1β* in the lungs in a combined model of traumatic brain injury model with acute EI (18). Similar results were confirmed in the liver as well, under the influence of an acute EI after HS in rats (3). In contrast to the anti-inflammatory effects of an acute EI, Hu et al. showed in their rat model of HS that an acute EI increased circulatory TNF-α levels and histopathological damage of the lung (39). The reports must be carefully interpreted since, e.g., Hu et al. administered EtOH intravenously (39), which is a physiologically absolutely different approach. Regarding the TNF-α levels in the serum, which we did not assess here, it is already well-reported that alcohol can induce the TNF-α release from the liver macrophages, the Kupffer cells, due to the LPS-driven NF-κB signaling pathway (41). Additionally, ethanol sensitizes the TNF receptor 1 (TNFR1) to the TNF, strengthening the inflammatory response and the recruitment of leukocytes (42), and thus contributing to local hepatic inflammation, as well as systemic inflammation. This could be a prevalent factor contributing to the circulating TNF-α levels; however it is worth mentioning that the macrophages’ general capability to express TNF-α is relatively low according to the gene/protein expression data from the Human Protein Atlas (ID: ENSG00000232810-TNF). Moreover, soluble TNF-α cytokine lifespan is rather short (43), which always brings difficulties as we have also observed in our study measuring the total circulating level of this cytokine and reflecting its importance more as a local mediator/attractant. Regarding the lung tissue response and production of TNF-α, ethanol brings mixed conclusions regarding its influence on cytokine production, impairing the LPS-driven TNF-α release and contributing to the expression of anti-inflammatory cytokines (44). We have observed this trend in our studies involving the sera derived from traumatic patients and co-incubated with human immortalized epithelial lung cells prior to the exposure to ethanol, which concluded in decreased activation of the NF-κB pathway and the desensitization of neutrophils (45). It is difficult to clearly affirm or denounce the elevated levels of circulating TNF-α coming from the Kupffer cells’ activation in the liver due to alcohol activity, while facing the difficulties described above; however, it is certain that the TNF-α gene expression levels change locally in the lung along the applied conditions. On the other hand, regarding the TNF-α levels, the disintegrin and metalloprotease 17, known also as ADAM-17 or TACE, is an enzyme capable of proteolytic cleavage of the TNF-α precursor protein, a membrane-anchored pro-protein, thus releasing the outer domain (known as ectodomain) into the mature, soluble TNF-α cytokine in the process known as “shedding” (46). This strongly indicates the role of ADAM-17 and other potentially “shedding” proteins in the regulation of expression levels of the final extracellular concentration of active TNF-α cytokine, as the target gene expression levels do not indicate the final levels of protein after translation, especially with secondary post-translational regulators such as ADAM-17, required for the maturation step of the protein. In our experimental setting, it is obviously a relevant factor for consideration, as it was shown by DeBerge et al. that the inhibition of ADAM-17 yields a decreased ratio of TNF-α processing in the animal model of lung injury and thus decreased levels of active cytokine and attenuated severity of lung injury and increased survival of mice (47). Moreover, what is especially relevant in our study is the observation that acute ethanol exposure is capable of regulating the expression of ADAM17 and thus downregulating the maturation of soluble TNF-α and its release (48, 49). However, downregulation of ADAM17 by ethanol does not exclude the negative influence of ethanol on the TNF-α gene expression, which appears to be the prevalent mechanism of action. Further comparisons were used to experimentally validate our present study. Acute EI reduced THFx-induced CXCL1 as well as PMNL presence in the lungs after THFx, which supports our previous reports obtained from the liver analyses upon an acute EI in HS (3). However, Sears et al. demonstrated an enhanced inflammatory response concomitant with increased leukocyte infiltration in lungs 24 h after acute EI and bilateral femur fracture in rats (40). These paradoxical observations might be explained in part by the differences in basic methodology. In contrast to our THFx (osteotomy) model with intragastric EtOH application, Sears et al. applied a blunt guillotine to induce femur fractures and injected EtOH intraperitoneally. In our scenario, acute EI significantly reduced uncontrolled lung inflammatory response and apoptosis after THFx. Acute EI had a marked impact on the Wnt/β-catenin signaling pathway by reducing THFx-induced *Wnt3a*, increasing sclerostin levels, and increasing β-catenin as demonstrated by the IF. Further studies are necessary to determine if increased sclerostin inhibits the active Wnt/β-catenin signaling pathway, ultimately increasing β-catenin in membranous complexes, and thus promoting the stability of adherens junctions after THFx as postulated here. To the best of our knowledge, there are no currently published studies that examine the influence of an acute EI on Wnt/β-catenin signaling pathway in the context of lung barrier breakdown after THFx. However, Dogget and Breslin investigated the impact of an acute EI on microvascular leakage in rats and found an increase in mesenteric microcirculation permeability caused by disrupted VE-cadherin organization at junctions (50). While our data indicate lung barrier stabilization, alcohol-induced endothelial barrier dysfunction was proposed by Dogget and Breslin (50). The ubiquitin-binding protein A20 was indispensable for regulating vascular E-cadherin expression in adherens junctions to maintain and repair damaged endothelial functions after pulmonary vascular injury by lipopolysaccharide (LPS) (11). According to Nakamura et al., it is presumed that A20 interacts with a proximal signaling protein RIPK4 of the Wnt signaling pathway through its natural affinity towards RIPK family proteins, and the regulation of Wnt signaling by A20 occurs *via* RIPK4, which interestingly enough acts by itself as a positive regulator of Wnt/β-catenin canonical signaling through its kinase activity of the Dsh protein (12). It is known that A20 negatively regulates the transcription factor of the nuclear factor kappa-light-chain-enhancer of activated B cells (NF-κB), i.e., through interacting with RIPK proteins, and it was shown that A20 is capable of suppressing the activation of the NF-κB in the experimental setting of the murine traumatic bone injury model, challenged with LPS as a stimulus (51). However, the data on A20 and its modulatory involvement in trauma with/without EI are still missing to a large extent. What we have observed in our study is presumably a potential correlation between those two factors, as both A20 and RIPK4 levels seem to change in a similar manner simultaneously, which may or may not imply a potential complexing interaction or molecular connection, which could be in line with the observations considering the role of A20 in interacting with RIPK proteins. We noticed that increased levels of both A20 and RIPK4 are connected to suppressed activity of Wnt/β-catenin signaling, while its downregulation by trauma injury only seems to induce the further release of β-catenin. This initial observation is thus contradictory to the data from Nakamura et al., as diminished levels of RIPK4 negatively impact the Wnt/β-catenin signaling, as it prohibits sequestration of the destruction complex (12). This may underline the role of A20 as a negative inhibitor of RIPK4, and it is A20 that is one of the targets of EI and trauma stimulation. We illustrate the potential interaction in **Figure 7**; however, it is clearly too early to draw any significant conclusions based only on those data. This aspect requires more insight and deep experimental investigations, especially on the protein–protein interaction plane with huge emphasis on studying the expression patterns.

Here, we show that acute EI prior to THFx downregulated the THFx-induced increase in Wnt3a gene expression, which correlated with an increased β-catenin expression in the lung. Thus, we hypothesize that the data indicate an inhibitory effect of acute EI on Wnt signaling in the lung after trauma. It appears that this effect either is organ-specific or depends on the type of alcohol intoxication (acute vs. chronic EI). Considering the effects of chronic alcohol abuse on Wnt signaling in, e.g., liver, there are several studies that report an opposite and, thus, enhancing impact of alcohol on Wnt signaling. The consequence of such impact is often enhanced development or progression of alcoholic liver disease (ALD), which can ultimately lead to hepatitis, fibrosis, cirrhosis, or hepatocellular carcinoma (52–54). Mercer et al. examined the effects of chronic alcohol abuse (16 weeks) on the development of diethylnitrosamine (DEN)-induced hepatic tumors in a mouse model (52). In tumor-free liver tissue from EtOH+DEN-treated mice, they found significantly increased β-catenin expression in the membrane and cytosol, and enhanced accumulation in the nucleus. Moreover, induction of alcohol-induced liver disease in rats resulted in increased expression of cytosolic and nuclear β-catenin, phosphorylated GSK3β, and significantly increased gene expression of Wnt2, Wnt7a, and β-catenin target genes (52). The consequence of increased Wnt signaling was a significantly increased incidence of liver tumors in the EtOH+DEN group compared to the controls (52). Similar effects were observed by Warner et al., who investigated the role of specific dietary fatty acids (n3-PUFA) in Wnt signaling and the development of ALD in mice (53). Wild type (WT) and fat-1 transgenic mice underwent chronic EI (6 weeks) with a single final LPS challenge. Chronic EI resulted in increased gene expression of Wnt5a and Wnt4 and significantly reduced gene expression of the negative feedback regulator of Wnt signaling Axin2 in WT mice compared with control animals without long-term alcohol consumption (53). The reported data contrast with our observed effects of acute EI on Wnt signaling in the lung, although it should be noted that our work involved acute EI and not long-term alcohol abuse, and that the influence of trauma was not examined in the above-mentioned studies. Additionally, it should be noted that studies on the influence of alcohol on Wnt signaling in the liver are inconclusive. Several studies exist that describe an inhibitory influence of chronic alcohol abuse on Wnt signaling. Huang et al. investigated the influence of pharmacological activation of Wnt signaling on ALD development and progression in a rat model (54). Rats were subjected to 8 weeks of alcohol consumption. The impact of a 3-week treatment with Wnt agonists in parallel to chronic EI was additionally investigated. They found decreased cytosolic and nuclear β-catenin expression in the liver of animals with chronic alcohol abuse compared with controls (54). Pharmacological activation of the Wnt signaling pathway resulted in decreased ALD progression (54). Similar effects were demonstrated in the work of Xu et al. who examined the crosstalk of chronic alcohol abuse and both the insulin/IGF and Wnt pathways on liver regeneration. After 8 weeks of alcohol administration, rat liver tissue showed decreased gene expression of Wnt1, Wnt7a, and Fzd3 compared to control animals without alcohol administration (55). These data also suggest inhibition of Wnt signaling by chronic alcohol abuse.

Despite the fact that the data suggest the Wnt signaling pathway being involved in the observed changes, there are definitely other involved mechanisms of the widely spoken multicellular impact of ethanol, independent of Wnt signaling, as ethanol influence is exceptionally broad and affects a magnitude of several dependent and independent physiological aspects (56–58), with many of them already settled down in our scientific interests (59, 60). In the study of Samuelson et al., the authors have shown an interesting result derived from an experimental model of the recolonization of microbiota from the alcohol-fed mice donors, followed up by the evaluation of lung inflammation severity and intestinal integrity upon *Klebsiella pneumoniae* infection (61). The study underlines the role of alcohol-induced dysbiosis in the pathogenesis of inflammation and the importance of gastrointestinal tract homeostatic microbiota in supporting the host defense capabilities, showing increased susceptibility to pneumonia severity in the alcohol-affected microbiota (61). The nature of *K. pneumoniae* infection is a key factor here, considering that as an opportunistic, Gram-negative bacteria species, *K. pneumoniae* releases LPS (endotoxin) to its environment, which leads to the activation of Toll-like receptors (TLRs), and thus transduction of proinflammatory transcription factors and the recruitment of inflammatory cells in the end (62). Moreover, there are lines of evidences that the LPS induction of TLR4 can activate the canonical Wnt/β-catenin signaling pathway (63), which further strengthens the significance of this pathway in ethanol-mediated mechanism of action. In regard to the work of Samuelson et al. once more, we had investigated the influence of alcohol on the integrity of intestinal barrier and observed in samples derived from the healthy volunteers after acute alcohol consumption early increased levels of circulating fatty acid binding protein (FABP-I), which acts as a biomarker for intestinal damage (64). This only further underlines the role and mechanisms of malfunctioning microbiota homeostasis in contributing to the pathogenesis of inflammation. Additionally, in this study, we observed the decreased levels of proinflammatory cytokines from trauma alcohol-fed mice, which stands in opposite to the data from severely affected and overgrown microbiota due to binge-on-chronic alcohol intake, where the proinflammatory cytokine levels were increased due to alcohol dysbiosis, further showing its effect on the enhanced inflammation (61). As indicated in **Figure 6**, the infiltration with other inflammatory cells such as CD4-positive T cells with the trauma-induced interferon-gamma also increases, and its decrease upon acute EI after trauma underlines the importance of other immune cells and pathways in the alcohol-modulated inflammation. In summary, there are definitely other mechanisms of ethanol action, where it displays its nature from both pro-inflammatory and anti-inflammatory capabilities, especially interplaying with traumatic injury and alcohol consumption. In this study, our focus was to investigate the DAMP-induced activation of immune response; however we are absolutely aware of the PAMP and gut–liver axis importance in the pathomechanism of damage, and we consider GI tract integrity as an important inflammatory benefactor during traumatic injury.

In our work, we observed that animals with acute EI before THFx exerted less lung injury. In contrast to the acute EI we studied, chronic EI is known to have adverse effects on the lung barrier. When alcohol consumption shifts from moderate or binge drinking to chronic alcohol consumption, increased permeability of the cellular lung barrier has been observed in numerous studies. Fan et al. found a significantly increased permeability of the alveolar epithelial barrier caused by increased disruption of tight junctions upon chronic EI (65). A mechanistic study by Otis et al. on the development of ARDS in prior chronic EI showed increased permeability of the alveolar epithelia with enhanced incidence of pulmonary edema in mice (66). In contrast to the effects of acute EI as observed also in the present study, Smith et al. identified chronic EI as a risk factor for the development of ARDS in sepsis (67). The observed effects of chronic alcohol consumption on the lung barrier not only have been demonstrated *in vivo* but also have been part of human studies. Burnham et al. examined pulmonary permeability in subjects with chronic EI showing that even in the absence of symptoms, individuals with a history of chronic EI had increased baseline lung permeability compared with controls, which could prime for a severe course of ARDS in the setting of sepsis (68). The negative effects of chronic EI in the context of ARDS have also been described by Berkowitz et al., demonstrating that patients with chronic EI also had a threefold increased risk of developing pulmonary edema (69). In general, it remains important to understand the mechanistically different effects of acute versus chronic EI, yet in regard to the public health context, alcohol consumption is one of the leading causes of accidents and, according to several clinical studies, the percentage of alcoholized polytrauma patients is over 25% (70). Alcohol consumption leads to increased financial, time, and personnel burden, requiring additional routine and specific laboratory controls, with clinical imaging mostly of the head due to the reduced vigilance, difficult anamnesis, and compliance of the patients admitted to hospitals (71).

The present study has its limitations. Most notably, we only used young male mice in our experiments. Thus, the conclusions can only be drawn for a younger patient cohort. Further research will be required to determine if the differences in age or gender correlate to the observed results. Additionally, some of the variability in the dataset may be caused by the sample size of mice. While the clinical scenario implies first fracture and then its stabilization, in this experimental model, an external fixator is provided first, and then the osteotomy. Femur fracture is frequently accompanied by hemorrhage, which underlines the importance of this model; however, the impact of isolated fracture on remote organ injury cannot be assessed by this model. To ensure comparability, we performed a controlled HS, which would not occur in a real trauma situation of uncontrolled bleeding. During the experiment, the animals were not ventilated using intubation, which might also influence the dataset. Interindividual differences regarding the absorption rate of EtOH may have an impact on the BAC; thus, intravenous administration of EtOH might improve the inter-group comparisons, as it was done in other studies (39). However, the intravenous EtOH application does not mimic real-life conditions, and reproducing the findings in the future using an alcohol feeding model, which may prove to have different exposure parameters due to the definition of “acute”, should be considered in translational models of EI. No conclusions can be drawn regarding the influence of subacute or chronic EI from this dataset. Also, in our work, we assessed the CXCL1 gene expression and protein expression in lung and BALF, and based on known data, and because of this, a subsequent increase in neutrophil invasion into lung tissue after THFx is observed. Together with known data, our results clearly indicate a CXCL1-mediated recruitment of neutrophils to lungs after trauma; however, in future studies, neutrophil counts in the BALF should be analyzed. Finally, the results of all the analyses are at a single time point in the complex process of THFx. Having multiple post-trauma time points would have allowed for a temporal analysis of EI-induced effects and mechanisms.

In summary, the current work adds novel information about the effects of canonical Wnt/β-catenin signaling pathway in trauma-induced uncontrolled local inflammatory response and lung injury. An acute EI alleviates the uncontrolled inflammatory response and lung barrier breakdown after trauma by suppressing the Wnt/β-catenin signaling pathway. Future studies should explore the association between acute EI and other upstream and downstream factors involved in Wnt/β-catenin signal transduction after trauma to provide interventional targets.

## Data Availability Statement

The data can be obtained upon a reasonable request from the corresponding author.

## Ethics Statement

This study was authorized by the local institutional animal care and research advisory committee and permitted by the local government of Lower Saxony, Germany (approval number: 33.12-42502-04-17/2491).

## Author Contributions

Conceptualization: CN and BR. Methodology: LN, BX, KB, YZ, and KK. Validation: AJN, LN, and BR. Formal analysis: LN and BR. Investigation: LN, BX, KB, SG, and AJN. Resources: CN and BR. Data curation: LN, BX, YZ, and BR. Writing—original draft preparation: LN, AJN, and BR. Writing—review and editing: KB, YZ, PJ, and CN. Visualization: BR. Supervision: CN, AJN, and BR. Funding acquisition: CN and BR. All authors contributed to the article and approved the submitted version.

## Funding

This research was funded by the German Research Foundation DFG with grant numbers DFG RE 3304/9-1, NE 1932/1-3, and 361210922/RTG 2408.

## Conflict of Interest

The authors declare that the research was conducted in the absence of any commercial or financial relationships that could be construed as a potential conflict of interest.

## Publisher’s Note

All claims expressed in this article are solely those of the authors and do not necessarily represent those of their affiliated organizations, or those of the publisher, the editors and the reviewers. Any product that may be evaluated in this article, or claim that may be made by its manufacturer, is not guaranteed or endorsed by the publisher.
